# Mineralocorticoid effects of fludrocortisone and hydrocortisone in primary adrenal insufficiency: EU-AIR patient data

**DOI:** 10.1007/s40618-025-02657-7

**Published:** 2025-09-06

**Authors:** Bertil Ekman, Marcus Quinkler, Pinggao Zhang, Andrea M. Isidori, Robert D. Murray, Jeanette Wahlberg

**Affiliations:** 1 Department of Endocrinology and Department of Health, Medicine and Caring Sciences, Linköping University, Linköping, Sweden; 2 Department of Internal Medicine and Department of Health, Medicine and Caring Sciences, Linköping University, Norrköping, Sweden; 3 Endocrinology in Charlottenburg, Berlin, Germany; 4 Takeda Development Center Americas, Inc, Cambridge, MA USA; 5 Department of Experimental Medicine, Sapienza University of Rome, Centre for Rare Diseases (Endo-ERN accredited), Policlinico Umberto I, Rome, Italy; 6 Department of Endocrinology, St James’s University Hospital, Leeds Teaching Hospitals NHS Trust, Leeds, UK; 7 School of Medical Sciences, Faculty of Medicine and Health, Örebro University, Örebro, Sweden

**Keywords:** Fludrocortisone, Glucocorticoid, Hydrocortisone, Mineralocorticoid, Primary adrenal insufficiency

## Abstract

**Purpose:**

Patients with primary adrenal insufficiency (PAI) require mineralocorticoid replacement therapy in addition to glucocorticoids. These therapies should be considered in combination because most glucocorticoids also possess mineralocorticoid activity. We aimed to investigate the relationship between fludrocortisone and hydrocortisone-equivalent dosing in patients with PAI.

**Methods:**

Data were obtained from the European Adrenal Insufficiency Registry (EU-AIR), a multinational, multicenter, observational study conducted between August 7, 2012, and October 31, 2020, in endocrinology centers in Germany, Italy, the Netherlands, Sweden, and the UK. Patients with PAI (excluding congenital adrenal hyperplasia or known hypertension) and treated with immediate-release hydrocortisone (IRHC), modified-release hydrocortisone (MRHC), or cortisone acetate were included. The relationship between hydrocortisone-equivalent and fludrocortisone doses and mineralocorticoid potency corrected for body surface area (BSA) was examined.

**Results:**

Overall, 670 (mean age: 46.2 years; 453 [67.6%] women) of 924 patients with PAI in EU-AIR were analyzed. Of those who received at least one dose of fludrocortisone (*n* = 350), 45 patients (12.9%) were receiving hydrocortisone-equivalent doses/BSA of ≤ 10 mg/day/m^2^, 170 patients (48.6%) > 10–15 mg/day/m^2^, and 133 patients (38.0%) > 15 mg/day/m^2^. No clear associations were found between total daily fludrocortisone dose/BSA and hydrocortisone-equivalent dose/BSA, or between combined mineralocorticoid potency/BSA and systolic or diastolic blood pressure and sodium or potassium levels. Higher systolic blood pressure was found in IRHC than MRHC groups.

**Conclusions:**

Fludrocortisone prescription in PAI appears to be independent of glucocorticoid replacement therapy. IRHC and MRHC might differ in mineralocorticoid effect owing to different pharmacokinetic profiles.

**Clinical trial registration:**

NCT01661387.

## Introduction

Primary adrenal insufficiency (PAI), also known as Addison’s disease, is a life-threatening disease [1, 2] with an estimated prevalence of 82–300 per 1 million in the general population [3–6]. All types of adrenal insufficiency (AI) result from impaired glucocorticoid secretion; however, unlike patients with secondary AI, those with PAI have an additional mineralocorticoid (MC) hormone deficit as a result of impaired aldosterone secretion [2, 7–9]. Therefore, patients with PAI require hormone replacement with both hydrocortisone and fludrocortisone (also called ‘9α-fluorohydrocortisone’ or ‘9α-fluorocortisol’) that act via glucocorticoid and MC receptors, respectively [8]. Current MC replacement consists of administering oral fludrocortisone 0.05–0.2 mg/day [1, 7, 9]. Typically, a dose of fludrocortisone 0.1 mg is given once daily in the morning, as physiological aldosterone secretion follows a circadian rhythm similar to that of cortisol, with peaks at 08:00 h and nadirs at 23:00 h [1, 8].

Reduced aldosterone secretion can result in clinical signs and symptoms of MC deficiency, such as hypotension, weakness, salt craving, and electrolyte disturbances including hyperkalemia and hyponatremia [9]. Additionally, the use of medications like diuretics and nonsteroidal anti-inflammatory drugs or licorice (an inhibitor of 11β-hydroxysteroid dehydrogenase type 1 and type 2) can affect blood pressure and electrolyte levels, and may necessitate dose adjustments of fludrocortisone [1, 10, 11]. Adrenal crises, which can occur as a result of inadequate glucocorticoid and MC replacement, are common in AI; a prospective study found a frequency of 8.3 crises per 100 patient-years in patients with AI, of which 6.3% were fatal [12]. Hence, either the over- or under-dosing of fludrocortisone may be linked to the increased mortality and morbidity, including adrenal crises, observed in PAI [1, 13–16].

Some glucocorticoids, including hydrocortisone and cortisone acetate, possess a degree of MC activity. It can be estimated, based on published calculations, that the MC activity of a standard dose (20 mg) of hydrocortisone is equivalent to that of 0.05 mg fludrocortisone [9, 17, 18]. Although the relationship between the hydrocortisone-equivalent dose and the MC activity may not be linear, it has been suggested that patients with PAI receiving low doses of hydrocortisone may require high doses of MC replacement therapy and vice versa [8, 9].

This analysis aimed to investigate the association between hydrocortisone-equivalent doses (including modified-release hydrocortisone [MRHC]) and fludrocortisone doses received by patients with PAI, using data from the European Adrenal Insufficiency Registry (EU-AIR).

## Materials and methods

### Study design and population

EU-AIR was a prospective, multinational, multicenter, observational study that was conducted between August 7, 2012, and October 31, 2020, to monitor the long-term safety of MRHC and conventional glucocorticoid replacement therapy during routine clinical practice in patients with AI (ClinicalTrials.gov: NCT01661387). All patients with a diagnosis of chronic AI (primary or secondary, or congenital adrenal hyperplasia [CAH]) receiving long-term glucocorticoid replacement therapy were eligible for inclusion in the registry.

Data were collected from endocrinology centers in Germany, Italy, the Netherlands, Sweden, and the UK [19]. Comprehensive data for patients with a diagnosis of primary or secondary AI or CAH were collected at baseline. This analysis included all patients with PAI treated with or without fludrocortisone (excluding those with hypertension or CAH) receiving immediate-release hydrocortisone (IRHC), MRHC, or cortisone acetate. All enrolled patients were followed up during routine clinical practice for the active duration of the registry. All medical care decisions, including those relating to treatment choice, were entirely at the patient’s and registry physician’s discretion. Patient data, including laboratory assessments, were collected using an electronic case report form at enrollment and during routine clinic visits (approximately every 6 months) thereafter. Before including patients in the registry, study personnel were provided with training on all aspects of the study. Clinics were provided with instructions on which variables to record and how to record them in the registry. Study scales were calibrated, and laboratory analyses were conducted according to standard practice. Data entered into the electronic case report form were checked automatically using logic checks and limits. Review and validation were performed to detect inconsistencies or missing data. If any confounding or missing data were detected, a data query was generated within the database for answering by clinic staff.

Hydrocortisone-equivalent doses of cortisone acetate were calculated by multiplying the dose of cortisone acetate by 0.8 [18]. Patients being treated for hypertension and patients receiving fludrocortisone doses greater than 300 µg were excluded owing to the potential influence of these factors on the normal MC balance. Using published data from Grossmann et al. regarding MC potency of various glucocorticoids [18], the combined MC potency of fludrocortisone + hydrocortisone-equivalent doses was calculated as described below and in Table 1.

**Table 1:** MC potency calculations for fludrocortisone and hydrocortisone

Glucocorticoid	MC potency factor [18]	MC potency
Fludrocortisone 0.1 mg	10	1
Hydrocortisone 10 mg	0.054	0.54
Hydrocortisone 20 mg	0.054	1.08
Hydrocortisone 30 mg	0.054	1.62

MC, mineralocorticoid

- Aldosterone (as a reference) has an MC potency factor of 1.
- Fludrocortisone has an MC potency factor of 10.
- Hydrocortisone has an MC potency factor of 0.054 (185 times lower than fludrocortisone).

*Examples*.

- A patient receiving fludrocortisone 0.1 mg and hydrocortisone 10 mg: 1 + 0.54 = 1.54 total MC potency.
- A patient receiving fludrocortisone 0.1 mg and hydrocortisone 20 mg: 1 + 1.08 = 2.08 total MC potency.
- A patient receiving fludrocortisone 0.15 mg and hydrocortisone 25 mg: 1.5 + 1.35 = 2.85 total MC potency.

Patients were divided into three groups based on the hydrocortisone-equivalent daily dose (corrected for body surface area [BSA]) that they were receiving: ≤ 10 mg, > 10–15 mg, and > 15 mg. The analysis examined the association between fludrocortisone dosing and hydrocortisone-equivalent dosing (including MRHC). Patients were excluded from this analysis if they did not have at least 366 days of follow-up from baseline or if they were exposed to fludrocortisone dosing and/or hydrocortisone-equivalent dosing for less than 274 days. Patients exposed for less than 274 days to both fludrocortisone dosing and hydrocortisone-equivalent dosing were included if they took either of these medications for at least 274 days.

### Statistical analysis

Descriptive statistics were used to analyze the data, including the number and percentage of observations, median, mean, and standard deviation (SD). Analysis of covariance (ANCOVA) was carried out to assess the influence of age, sex, body mass index (BMI), and MC potency/BSA on diastolic and systolic blood pressure, and sodium and potassium levels. Differences were considered statistically significant when *p* ≤ 0.05. In addition, the Student’s t-test and Fisher’s exact test were used to compare MRHC and IRHC for continuous and binary confounding factors, respectively.

### Ethical approval

This study was approved by the appropriate local research ethics committees for all participating centers and was conducted in accordance with the Declaration of Helsinki. Each patient and/or their parent/legal guardian provided written informed consent before enrollment in EU-AIR.

## Results

Between August 7, 2012, and October 31, 2020, 3247 patients were enrolled in EU-AIR (MRHC: *n* = 645; other glucocorticoid replacement therapies: *n* = 2843; some patients received both MRHC and other glucocorticoid replacement therapies). Of these patients, 924 had PAI. In total, 254 patients were excluded from the analysis owing to adverse events (*n* = 127), hypertension (*n* = 12), or receiving medication for hypertension (*n* = 178), medication for fewer than 274 days (*n* = 50), or other glucocorticoids (*n* = 92). Altogether, 670 patients were analyzed. Overall, 605 patients with PAI received at least one dose of fludrocortisone, and 65 patients received only hydrocortisone. Most patients were women (*n* = 453, 67.6%), and the mean ± SD age was 46.2 ± 15.4 years (Table 2). The majority of patients (57.3%) had a BMI of less than 25 kg/m^2^ and 14.9% had a BMI of 30 kg/m^2^ or more. In total, 193 patients received MRHC and 475 patients received IRHC (Table 3). The median fludrocortisone daily dose received was 100 µg both in patients receiving MRHC and those receiving IRHC/cortisone acetate. It should be noted that the BSA data, which were used to correct dosing, were collected from 375/670 patients included in the analysis.

**Table 2:** Baseline demographics and characteristics of patients with PAI and without hypertension^a^

Parameter	Analysis population (*N* = 670)
Age, years, mean ± SD	46.2 ± 15.35
Weight, kg, mean ± SD	73.4 ± 17.20
Height, cm, mean ± SD	169.4 ± 10.28
Men, n (%)	217 (32.4)
Women, n (%)	453 (67.6)
BMI, kg/m^2^, mean ± SD	*n* = 375 25.6 (5.34)
BMI (kg/m^2^) category, n (%)	*n* = 375
Underweight (< 18.5)	9 (2.4)
Normal (18.5–< 25)	206 (54.9)
Overweight (25–< 30)	104 (27.7)
Obese (≥ 30)	56 (14.9)
BSA, m^2^, mean ± SD	*n* = 375 1.8 ± 0.23
MC potency, mean ± SD	*n* = 668^*b*^ 2.6 ± 1.40
MC potency/BSA, mean ± SD	*n* = 375 1.5 ± 0.78
Systolic blood pressure, mmHg, mean ± SD	*n* = 453 124.5 ± 17.64
Diastolic blood pressure, mmHg, mean ± SD	*n* = 454 77.7 ± 10.35

BMI, body mass index; BSA, body surface area; MC, mineralocorticoid; PAI, primary adrenal insufficiency; SD, standard deviation

^a^All patients were receiving fludrocortisone with immediate-release hydrocortisone, modified-release hydrocortisone, or cortisone acetate

^b^Two patients with MC potency values of 301 and 101 were excluded from this analysis

**Table 3:** Comparison of baseline demographics in patients with PAI receiving MRHC versus IRHC

Parameter	Fludrocortisone + hydrocortisone		Hydrocortisone only	
	MRHC (*n* = 178)	IRHC (*n* = 425)	MRHC (*n* = 15)	IRHC (*n* = 50)
Age, years, mean ± SD	44.6 ± 13.83	47.0 ± 15.93	44.3 ± 11.91	46.1 ± 16.30
Below 40 years of age, n (%)	67 (37.6)	141 (33.2)	5 (33.3)	18 (36.0)
Men, n (%)	54 (30.3)	140 (32.9)	4 (26.7)	18 (36.0)
Women, n (%)	124 (69.7)	285 (67.1)	11 (73.3)	32 (64.0)
BSA, m^2^, mean ± SD	*n* = 123 1.8 ± 0.18	*n* = 225 1.8 ± 0.23	*n* = 9 1.8 ± 0.26	*n* = 18 2.0 ± 0.33
MC potency, mean ± SD	*n* = 178 3.0 ± 1.78	*n* = 423 2.6 ± 1.16	*n* = 15 1.5 ± 0.76	*n* = 50 1.2 ± 0.73
MC potency/BSA, mean ± SD	*n* = 123 1.7 ± 0.95	*n* = 225 1.4 ± 0.64	*n* = 9 0.8 ± 0.34	*n* = 18 0.6 ± 0.26
Systolic blood pressure, mmHg, mean ± SD	*n* = 135 119.7 ± 15.90	*n* = 284 126.4 ± 18.00	*n* = 9 117.7 ± 9.38	*n* = 24 130.1 ± 18.54
Diastolic blood pressure, mmHg, mean ± SD	*n* = 135 76.2 ± 10.42	*n* = 285 78.4 ± 10.43	*n* = 9 77.8 ± 9.38	*n* = 24 77.0 ± 8.46
Potassium, mmol/L, mean ± SD	*n* = 129 4.2 ± 0.40	*n* = 260 4.3 ± 0.42	*n* = 10 4.3 ± 0.40	*n* = 18 4.3 ± 0.45
Sodium, mmol/L, mean ± SD	*n* = 130 139.7 ± 2.38	*n* = 259 139.2 ± 2.64	*n* = 9 140.7 ± 1.73	*n* = 17 140.4 ± 3.08
Hydrocortisone daily dose,^a^ mg, mean ± SD	*n* = 178 34.3 ± 25.57	*n* = 425 24.7 ± 10.65	*n* = 15 27.2 ± 14.16	*n* = 50 22.4 ± 12.65
Fludrocortisone daily dose, mg, mean ± SD	*n* = 178 0.1 ± 0.11	*n* = 425 0.2 ± 1.53	*n* = 15 0	*n* = 50 0

BSA, body surface area; IRHC, immediate-release hydrocortisone; MC, mineralocorticoid; MRHC, modified-release hydrocortisone; PAI, primary adrenal insufficiency; SD, standard deviation

^a^Hydrocortisone-equivalent dose

### Hydrocortisone doses

The mean hydrocortisone-equivalent dose (mg/day) ± SD was 27.1 ± 16.8, whereas the hydrocortisone-equivalent dose/BSA (mg/day/m^2^) ± SD was 16.0 ± 10.5. The numbers of patients receiving hydrocortisone-equivalent doses/BSA of > 10–15 mg/day/m^2^ (170 [48.6%]) and > 15 mg/day/m^2^ (133 [38.0%]) were numerically higher than those receiving doses ≤ 10 mg/day/m^2^ (45 [12.9%]). The mean dose ± SD of fludrocortisone/BSA was similar across the three patient groups, with a tendency toward higher fludrocortisone doses in the ≤ 10 mg/day/m^2^ group (70 ± 54 µg/day/m^2^) than in the groups receiving hydrocortisone-equivalent doses/BSA of > 10–15 mg/day/m^2^ (64 ± 56 µg/day/m^2^) or > 15 mg/day/m^2^ (61 ± 42 mg/day/m^2^).

### MC potency

Mean MC potency ± SD was 2.6 ± 1.40 (Table 2). MC potency/BSA ± SD was 1.5 ± 0.78, and 278 patients (41.5%) had an MC potency greater than 2.5.

### Correlations between hydrocortisone and fludrocortisone doses

**Fig. 1:**
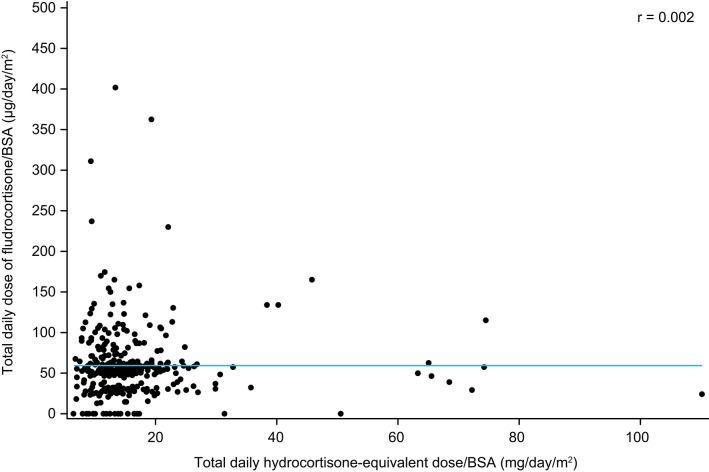
Correlation between the total daily hydrocortisone-equivalent dose/BSA and the total daily fludrocortisone dose/BSA taken by patients with PAI. BSA, body surface area; PAI, primary adrenal insufficiency

**Fig. 2:**
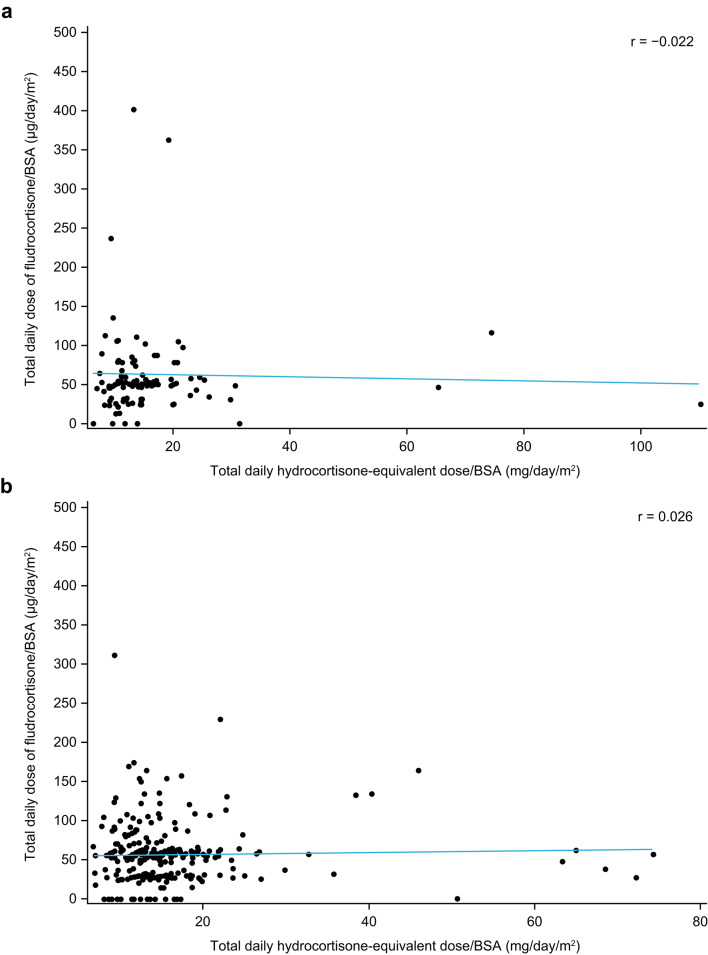
Correlation between the total daily hydrocortisone-equivalent dose/BSA and the total daily fludrocortisone dose/BSA taken by (**a**) men and (**b**) women with PAI. BSA, body surface area; PAI, primary adrenal insufficiency

Overall, there was no correlation between the total daily hydrocortisone-equivalent dose/BSA and the total daily dose of fludrocortisone/BSA received by patients with PAI (*r* = 0.002) (Fig. 1). Similarly, there was no association between hydrocortisone-equivalent and fludrocortisone doses/BSA taken by men (*r* = −0.022; Fig. 2a) or women (*r* = 0.026; Fig. 2b). Generally, there were more women than men receiving a total daily dose greater than 100 µg/day/m^2^ of fludrocortisone/BSA; however, no formal comparison was performed. The total daily dose of fludrocortisone/BSA was not associated with age (*r* = −0.139) or BMI (*r* = −0.082) in this cohort of patients with PAI.

### Correlations between MC potency/BSA and blood pressure or electrolytes

**Fig. 3:**
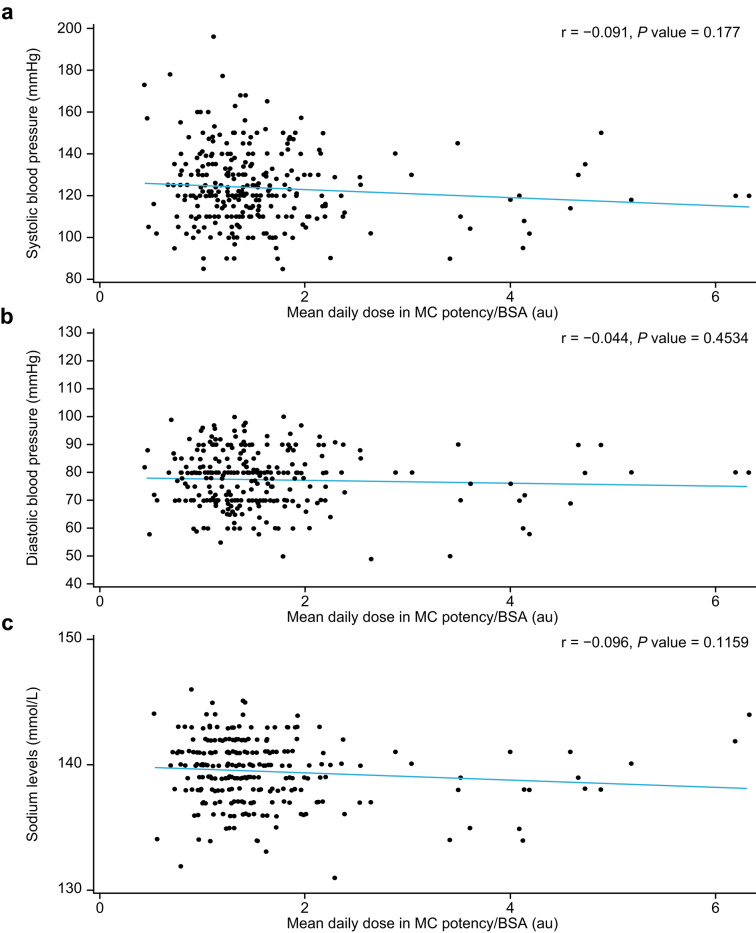

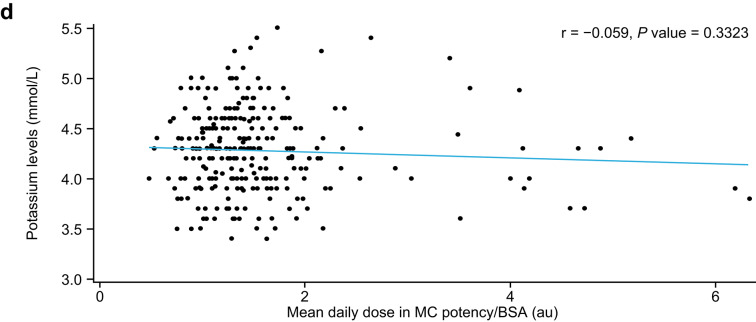
Relationship between MC potency/BSA and (**a**) systolic blood pressure, (**b**) diastolic blood pressure, (**c**) sodium levels, and (**d**) potassium levels in patients with PAI and without hypertension or using hypertensive medication. All patients were receiving fludrocortisone with immediate-release hydrocortisone, modified-release hydrocortisone, or cortisone acetate. AU, arbitrary unit; BSA, body surface area; MC, mineralocorticoid; PAI, primary adrenal insufficiency

There was no correlation between MC potency/BSA and systolic blood pressure, diastolic blood pressure, sodium levels, or potassium levels (Fig. 3). Overall, 190 patients (20.6%) with hypertension or taking medication for hypertension were excluded from the analysis. Despite this, blood pressure levels varied extensively, as shown in Fig. 3, and patients with PAI had a systolic blood pressure ranging from 85.0 to 196.0 mmHg (*n* = 534).

### Multivariate analyses

In the multivariate analyses, no relationships were found between MC potency/BSA and systolic blood pressure (F = 0.9803, *p* value for F-statistic [Pr > F] = 0.3228), diastolic blood pressure (F = 0.2048, Pr > F = 0.6512), sodium levels (F = 3.1035, Pr > F = 0.0791), or potassium levels (F = 1.4677, Pr > F = 0.2266) in patients with PAI. Systolic blood pressure was significantly positively correlated with age (F = 45.70, Pr > F = < 0.0001) and sex (F = 11.15, Pr > F = 0.0009; such that it was higher in men than in women). Similarly, diastolic blood pressure was significantly positively correlated with age (F = 13.29, Pr > F = 0.0003), but not with sex (F = 2.4589, Pr > F = 0.1177). Treatment type (MRHC vs. IRHC) was significantly correlated with systolic blood pressure (F = 9.4245, Pr > F = 0.0023; such that it was higher in IRHC than MRHC) and sodium levels (F = 4.5911, Pr > F = 0.0329).

### Comparison between IRHC and MRHC

**Fig. 4:**
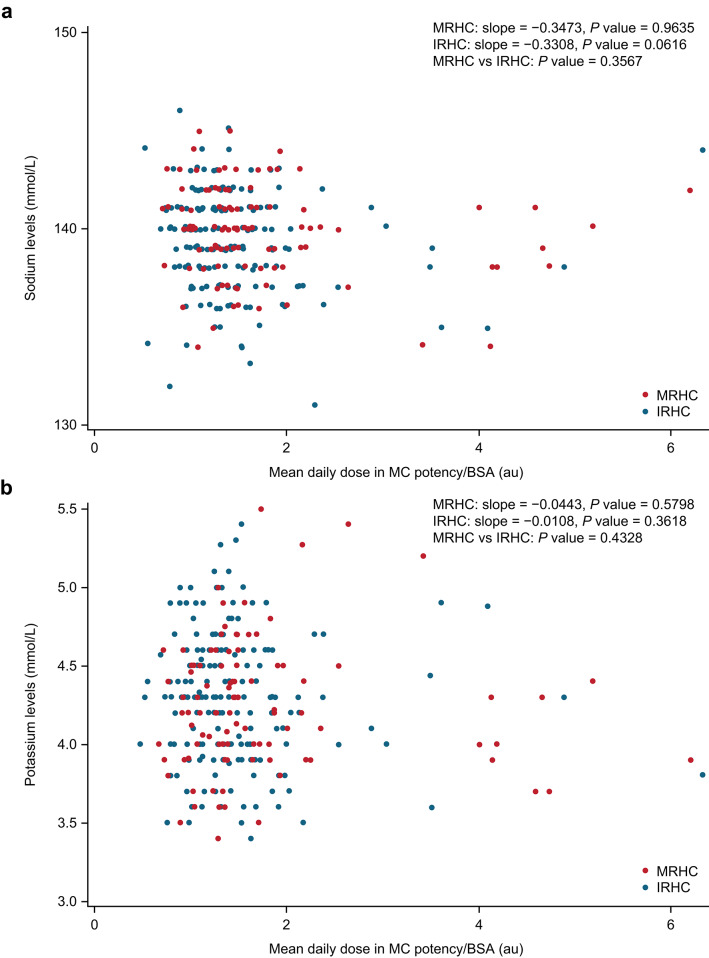
Relationship between MC potency/BSA and (**a**) sodium and (**b**) potassium levels in patients with PAI receiving IRHC or MRHC. AU, arbitrary unit; BSA, body surface area; IRHC, immediate-release hydrocortisone; MC, mineralocorticoid; MRHC, modified-release hydrocortisone; PAI, primary adrenal insufficiency

No differences in the association between MC potency/BSA and sodium levels (IRHC: slope = – 03308, *p* = 0.0616; MRHC: slope = −0.3473, *p* = 0.9635) or potassium levels (IRHC: slope = −0.0108, *p* = 0.3618; MRHC: slope = −0.0443, *p* = 0.5798) were found between patients with PAI receiving IRHC and those receiving MRHC (Fig. 4).

## Discussion

Fludrocortisone dosing for patients with AI has received relatively little attention in the literature compared with glucocorticoid dosing [20]. The current analysis investigated fludrocortisone dosing in a large population of patients with PAI, using data from EU-AIR, the largest multinational registry for patients with AI to date. The results of this descriptive analysis demonstrate that total daily doses of fludrocortisone received by patients with PAI (excluding those with CAH or known hypertension) vary widely, from no treatment at all to 0.3 mg, and are not associated with total daily hydrocortisone-equivalent doses/BSA. Therefore, it can be hypothesized that in some cases of PAI, varying degrees of residual aldosterone and/or cortisol secretion may be sufficient for some patients to avoid the MC deficiency that would otherwise require replacement therapy with fludrocortisone [21].

The absence of a strong association between fludrocortisone and hydrocortisone-equivalent doses suggests that physicians may consider the prescription of fludrocortisone independently of glucocorticoid replacement therapy. Fludrocortisone is indicated at a daily dose of 50–200 µg for MC replacement, with dose titrations based on the clinical evaluation of signs and symptoms of over- (e.g., edema and hypertension) and under- (e.g., hypovolemia, fatigue, feeling lightheaded and salt craving, and postural hypotension) replacement, on serum sodium and potassium levels, and plasma renin activity or concentration [1, 9, 22, 23]. In this study, data on plasma renin activity or concentration were not available owing to the lack of a standardized method of collecting and analyzing these data across study centers. Notably, in a study in patients with CAH and PAI, plasma renin concentration was not found to be correlated with MC dose; however, electrolyte levels were strongly correlated with MC dose [24]. More recently, potassium levels were identified as one of the few predictors of total daily MC dose in patients with PAI [25]. However, patients with salt-wasting CAH cannot be compared with patients who have autoimmune PAI because 21-hydroxylase deficiency in CAH presents with high levels of 17-hydroxyprogesterone, a known MC-receptor antagonist. The effects of 17-hydroxyprogesterone levels on the renin–angiotensin–aldosterone system, serum electrolytes, and blood pressure were recently reported by Tschaidse et al. in patients with salt-wasting CAH [26]. Furthermore, sex-related differences identified in the renin–angiotensin–aldosterone system may influence renal and cardiovascular physiology [27]. In patients with autoimmune PAI, it was shown that plasma sodium and potassium levels required only a small MC dosage for normalization, and in patients on higher fludrocortisone dosages, electrolyte levels exhibit only small changes. Once electrolyte levels had normalized, it was likely that they did not correlate further with increasing fludrocortisone doses.

Most patients in this study were receiving fludrocortisone doses within the expected range; however, a proportion of patients were receiving doses of less than 50 µg per day or no treatment. These results are in line with reports that MC under-replacement is common in patients with PAI [8]. Furthermore, assessing adherence to a prescribed MC regimen is a frequently neglected issue [25]. In a study of 22 patients with PAI, it was reported that the mean plasma volume was reduced to 87% of the predicted level based on patients’ height, weight, and sex, suggesting substantial hypovolemia despite MC and glucocorticoid replacement therapy [28]. Furthermore, a study of 15 patients with PAI suggested that the under-replacement of MCs may be compensated for by the over-replacement of glucocorticoids [29].

It is well established that patients with AI have a significantly reduced health-related quality of life and life expectancy compared with the general population [30–32]; for example, in Sweden, the standardized mortality rate has been reported as twofold greater for patients with PAI compared with the general population [31]. Neglecting optimal MC replacement could contribute to the failure of corticosteroid replacement therapy to prevent adrenal crises and restore health-related quality of life and/or life expectancy in patients with PAI. Conversely, exceedingly high doses of MC replacement therapy might result in high blood pressure, a higher risk of cardiovascular diseases, and higher morbidity and mortality [8, 14, 23, 33]. Furthermore, Skov et al. reported that the risk of cardiovascular disease was associated with higher MC replacement doses in women [33]. To investigate the MC effects of both glucocorticoid and MC replacement therapy in the present study, MC potency/BSA factor was calculated. However, a correlation between MC potency and systolic or diastolic blood pressure was not detected, indicating that there was no MC over-replacement in the patients analyzed.

To our knowledge, this is the first study to investigate possible differences in MC replacement therapy between IRHC and MRHC. MRHC tablets provide a more circadian-based serum cortisol profile than IRHC and may act differently in the body. In the original clinical study of MRHC with a crossover design, compared with the equivalent dose of IRHC, reduced body weight and reduced blood pressure were reported with MRHC treatment [34]. In line with these results, a difference between MRHC and IRHC was found in the correlation of MC potency/BSA and systolic blood pressure. An alternative explanation may be that high doses of IRHC result in high serum cortisol concentrations that might directly influence the arterial walls [35–37]. Therefore, MRHC may be beneficial for avoiding MC over-replacement.

Although these analyses were based on a large number of patients, especially considering the rarity of this disease, the lack of a clear correlation between MC potency/BSA and systolic blood pressure, diastolic blood pressure, and sodium and potassium levels is unexpected. These results could suggest the influence of extraneous factors that were not accounted for, such as volume status, body posture, temperature, salt intake, concomitant medications (e.g., nonsteroidal anti-inflammatory drugs) [11, 38, 39], and differences in plasma renin activity or concentration. Despite excluding patients with known hypertension, a large proportion of patients in the study population had elevated blood pressure (data not shown), indicating that mechanisms other than MCs (e.g., genetic predisposition) can affect blood pressure [40]. Possible further explanations may include excessive doses of glucocorticoids or mineralocorticoids, the age of patients, duration of the disease, or a secondary elevation of vasopressin (antidiuretic hormone) with fluid retention and vasoconstriction. Nevertheless, our results highlight the need for further research concerning fludrocortisone dosing in patients with PAI. Future work should focus on patients with high blood pressure who may have a high risk of cardiovascular diseases and those with low blood pressure who may be at risk of vascular dysregulation leading to or promoting the development of an adrenal crisis.

A limitation of the present study is the lack of a standardized protocol for assessing blood pressure. This was a real-world registry study, and not all potentially relevant data were available. For example, central blood analyses were not conducted at central reading centers, and data for plasma renin activity or concentration were not collected due to different protocols between centers. Indeed, the BSA data that were used to correct dosing were collected from 56.0% of patients in this analysis. Moreover, the fact that patients with PAI and known hypertension were excluded may have led to the exclusion of older patients who may have received lower fludrocortisone doses. Furthermore, the relationship between hydrocortisone dose and presumed MC activity, particularly for low doses of hydrocortisone, is not linear owing to protection of the MC receptor from cortisol by 11β-hydroxysteroid dehydrogenase type 2 [41]. Therefore, low doses of hydrocortisone are likely to have minimal MC activity.

The results of the present study suggest that the prescription of fludrocortisone in patients with PAI is currently considered independently of glucocorticoid replacement therapy in real-world clinical practice. MRHC treatment resulted in lower systolic blood pressure and lower serum sodium concentrations than IRHC treatment independent of the fludrocortisone dose. Furthermore, it should be noted that even in a tertiary referral center a high proportion of patients with PAI had high blood pressure, which should be investigated further to avoid cardiovascular disease.

## Data Availability

Restrictions apply to the availability of some or all data generated or analyzed during this study to preserve patient confidentiality or because they were used under license. The corresponding author will, on request, detail the restrictions and any conditions under which access to some data may be provided.
